# The bZIP Transcription Factor LtAP1 Modulates Oxidative Stress Tolerance and Virulence in the Peach Gummosis Fungus *Lasiodiplodia theobromae*

**DOI:** 10.3389/fmicb.2021.741842

**Published:** 2021-09-23

**Authors:** He Zhang, Wanqi Shen, Dongmei Zhang, Xingyi Shen, Fan Wang, Tom Hsiang, Junwei Liu, Guohuai Li

**Affiliations:** ^1^Key Laboratory of Horticultural Plant Biology-Ministry of Education, College of Horticulture and Forestry Sciences, Huazhong Agricultural University, Wuhan, China; ^2^Haikou Experimental Station, Chinese Academy of Tropical Agricultural Sciences, Haikou, China; ^3^Jiangxi Oil-tea Camellia, Jiujiang University, Jiujiang, China; ^4^School of Environmental Sciences, University of Guelph, Guelph, ON, Canada

**Keywords:** AP1 transcription factor, fungal virulence, *Lasiodiplodia theobromae*, oxidative stress response, peach gummosis disease, plant defense response

## Abstract

*Lasiodiplodia theobromae* is one of the primary causal agents in peach gummosis disease, leading to enormous losses in peach production. In our previous study, a redox-related gene, *LtAP1*, from the fungus was significantly upregulated in peach shoots throughout infection. Here, we characterized *LtAP1*, a basic leucine zipper transcription factor, during peach gummosis progression using the CRISPR-Cas9 system and homologous recombination. The results showed that *LtAP1*-deletion mutant had slower vegetative growth and increased sensitivity to several oxidative and nitrosative stress agents. *LtAP1* was highly induced by exogenous oxidants treatment in the *L. theobromae* wild-type strain. In a pathogenicity test, the deletion mutant showed decreased virulence (reduced size of necrotic lesions, less gum release, and decreased pathogen biomass) on infected peach shoots compared to the wild-type strain. The mutant showed severely reduced transcription levels of genes related to glutaredoxin and thioredoxin in *L. theobroame* under oxidative stress or during infection, indicating an attenuated capacity for reactive oxygen species (ROS) detoxification. When shoots were treated with an NADPH oxidase inhibitor, the pathogenicity of the mutant was partially restored. Moreover, ROS production and plant defense response were strongly activated in peach shoots infected by the mutant. These results highlight the crucial role of *LtAP1* in the oxidative stress response, and further that it acts as an important virulence factor through modulating the fungal ROS-detoxification system and the plant defense response.

## Introduction

The necrotrophic fungus, *Lasiodiplodia theobromae*, is geographically widespread in the subtropical and tropical regions and is known to attack approximately 500 plant species, including crops and woody trees (Alves et al., 2008; Cipriano et al., 2015). This fungus has been regarded as a latent pathogen or an opportunistic pathogen leading to dieback, canker, or fruit rot diseases in many economically important woody crops (Slippers and Wingfield, 2007; Ali et al., 2019). In southern China, *L. theobromae* is also a causal agent of peach gummosis, one of the most devastating diseases of peach (*Prunus persica*), annually causing considerable quantity and quality losses (Beckman et al., 2003; Wang et al., 2011). A better understanding of the molecular mechanisms of the peach-*L. theobromae* interaction is necessary for effective control of peach gummosis.

To establish successful infections, pathogens need to overcome both preformed and induced host defenses (Qi et al., 2017). During pathogen attacks, one of the major and fastest plant defense responses is a rapid accumulation of reactive oxygen species (ROS) at the invasion site (Scheler et al., 2013). ROS, primarily superoxide ($O_{2}^{-}$) and hydrogen peroxide (H_2_O_2_), are produced by plasma membrane-localized NADPH oxidases, also known as respiratory burst homologs (RBOH), at the inoculation site (Suzuki et al., 2011). Due to the toxicity, ROS can cause oxidative stress and damage to biomolecules, such as DNA mutation, lipid peroxidation, and protein oxidation, eventually causing cell death of the pathogens (De Gara et al., 2003).

Additionally, as a class of signaling molecules, ROS play crucial roles in plant-pathogen interactions. Plant-derived ROS act as signaling molecules to mediate various important responses of plant cells to fight against pathogen infection and enhance plant resistance by inducing plant defense-related gene expression and activating related enzyme activity (Torres and Dangl, 2005; Baxter et al., 2014). Our previous study demonstrated that the infection by *L. theobromae* caused a ROS burst, and transcripts of pathogenesis-related (*PR*) genes were markedly induced, potentially contributing to the restriction of disease development (Zhang et al., 2020).

To survive and colonize under harsh conditions, pathogens have developed ROS scavenging systems to efficiently reclaim excess ROS (Segal and Wilson, 2018). Scavenging enzymatic and non-enzymatic compounds, either preformed or induced, include superoxide dismutase (SOD), catalases (CAT), peroxidases (POD), glutaredoxins, and thioredoxins (Kawasaki et al., 1997; Lanfranco et al., 2005; Ma et al., 2018). The glutaredoxin system has glutathione, glutathione peroxidase (GPX), glutathione reductase (GLR), and NADPH. The thioredoxin machinery includes thioredoxin peroxidase [equal to thiol-specific antioxidant protein (TSA)], thioredoxin reductase (TRR), thioredoxins (TRX), and NADPH (Ma et al., 2018; Zhang et al., 2019). In fungal pathogens, transcription factor-mediated ROS detoxification through the regulation of antioxidant genes expression is vital in plant-pathogen interactions. One of the critical regulators mediating ROS detoxification is the Activating Protein 1 (AP1) class of basic leucine zipper (bZIP) family (Segal and Wilson, 2018). AP1 is a key transcriptional activator in response to oxidative stress in yeasts and filamentous fungi (Reverberi et al., 2008; Lin et al., 2018; Segal and Wilson, 2018). In our previous study, the *LtAP1* gene was consistently and highly expressed in the infection stage of *L. theobromae* on peach shoots, implying that *LtAP1* may play a crucial role in the pathogenicity of *L. theobromae* (Zhang et al., 2020).

*Saccharomyces cerevisiae* YAP1 serves as one of the most critical determinants of yeast to oxidative stress response, which is responsible for transcriptional activation of various ROS detoxification-related genes (Mendoza-Martínez et al., 2020). Subsequently, YAP1 homologs in several fungal pathogens were identified and characterized, and found to have conserved roles in oxidative stress response and tolerance, but differed in virulence. YAP1-mediated ROS detoxification has been identified as being an essential virulence determinant in the necrotrophic fungus *Alternaria alternata* (Lin et al., 2009), the hemibiotrophic rice blast fungus *Magnaporthe oryzae* (Guo et al., 2011), and the biotrophic maize pathogen *Ustilago maydis* (Molina and Kahmann, 2007). However, YAP1-assisted ROS detoxification is associated with avirulence in the animal pathogen *Aspergillus fumigatus* (Lessing et al., 2007), the necrotrophic plant pathogen *Cochliobolus heterostrophus* (Lev et al., 2005), or the hemibiotrophic plant pathogen *Fusarium graminearum* (Montibus et al., 2013). Although many studies have examined YAP1 homologs in other fungi, their function in *L. theobromae* during pathogenesis remains poorly understood, particularly for canker or gummosis disease in woody fruit trees. Understanding the role of the *LtAP1* gene in *L. theobromae* may lead to new tools to develop novel, sustainable disease management strategies against peach gummosis.

In this study, transcription factor LtAP1 was isolated and functionally characterized through genetic transformation. We examined the effects of deletion of the *LtAP1* gene on mycelial growth, sensitivity to oxidative and nitrosative stresses, and pathogenicity. This study shed some light on the function of the *LtAP1* gene for ROS detoxification, virulence, and suppression of plant defense responses during *L. theobromae* and peach interaction, which could deepen our knowledge of the role of fungal YAP1s in plant diseases.

## Materials and Methods

### Fungal Strains, Culture Conditions, and Chemical Treatments

*Lasiodiplodia theobromae* pathogenic strain JMB122, obtained from a peach tree with gummosis in Hubei Province, China (Wang et al., 2011), was used as a recipient host for transformation experiments. Both JMB122 and its derivatives were cultured on PDA medium (200gL^−1^ potato, 20gL^−1^ dextrose, and 15gL^−1^ agar) in a growth chamber at 28°C for 36h under a 12h-light/12h-dark cycle to assess growth and colony characteristics.

To test stress treatments, the wild type (WT) and genetic transformants of JMB122 were cultured on PDA plates (diameter 9cm) containing various chemical reagents. The integrity of cell walls and cell membranes was examined on PDA supplemented with calcofluor white (CFW; 0.05mgml^−1^), Congo red (2.5mgml^−1^), or sodium dodecyl sulfate (SDS; 0.02%). For oxidative stress, PDA was amended with H_2_O_2_ (1 or 2.5mm), *tert*-butyl-hydroperoxide (TBHP; 0.5mm), cumene H_2_O_2_ (0.68mm), or menadione (0.1mm). For nitrosative stress, PDA was amended with sodium nitroferricyanide dihydrate (SNP; 5mm). PDA was supplemented with glucose (1M) or KCl (1 M) for osmotic stress. PDA without amendments was used as control. Mycelial plugs (5mm diameter) were removed from the edge of 2-day-old colonies of each isolate and placed hyphal side down into the center of PDA plates. After 36h, the colony diameter was measured using a digital caliper, with four measurements from each plate. The growth inhibition rate (%) was calculated using the following formula: (diameter of untreated colony grown on PDA – diameter of colony grown on PDA with inhibitor treatment)/ diameter of untreated colony grown on PDA×100%.

As for the NADPH oxidase inhibitor diphenylene iodonium (DPI), the *L. theobromae*-inoculated shoots were treated with 5ml of DPI [dissolved in dimethyl sulfoxide (DMSO) and then diluted with water] at a concentration of 0.4μm at 12 and 24h after inoculation. Some inoculated peach shoots were mock treated with 0.04% DMSO. All the assays were independently performed in triplicate.

### RNA Extraction, cDNA Synthesis, and Gene Expression

RNA extraction, cDNA synthesis, and gene expression were conducted following Zhang et al. (2020). The two genes, translation elongation factor 2 (*PpTEF2*; Gao et al., 2016; Zhang et al., 2020) and tubulin (*LtTUB*; Zhang et al., 2020), were used as internal standards to normalize gene transcripts of *L. theobromae* and peach, respectively. The primers used for quantitative real-time PCR (qRT-PCR) are detailed in Supplementary Table S1. The relative expression was calculated using the comparative 2^−ΔΔCT^ method (Livak and Schmittgen, 2001) and expressed as means ± SD. The experiments were conducted with three independent biological replicates, each with four technical replicates.

### Gene Cloning and Identification

For cloning and identification of *LtAP1*, the strain JMB122 was cultured on PDA plates for 36h, and then the hyphae were collected for genomic DNA extraction following Wang et al. (2011). The putative LtAP1 protein sequences were obtained using orthologs of AP1 protein sequences of *S. cerevisiae* (Kuge and Jones, 1994) and *M. oryzae* (Guo et al., 2011) as BLASTP queries against the *L. theobromae* genome assembly (Félix et al., 2019), and one putative LtAP1-encoding gene was obtained from the genome assembly of *L. theobromae*. To confirm the presence of *LtAP1* in JMB122, the full length of *LtAP1* was amplified by PCR with primers FD120/FD121 (Supplementary Table S1). Open reading frames (ORF) and exon/intron positions in *LtAP1* were determined by comparison with *LtAP1* genomic DNA and cDNA sequences.

The predicted LtAP1 protein sequences from JMB122 were used to find orthologs in GenBank. The protein sequences of LtAP1 and its orthologs from various fungal species were aligned using Clustal X 1.81 (Thompson et al., 1997), and then, a phylogenetic tree was constructed using the neighbor-joining method with 1,000 bootstrap replications in MEGA 6.0 software (Tamura et al., 2013).

### Targeted Gene Disruption

The *LtAP1* knockout transformants were obtained using homologous recombination and the CRISPR/Cas9 approach (Ma et al., 2018; Zhang et al., 2020). The upstream (1,688bp) and downstream (1,722bp) fragments of the *LtAP1* gene of strain JMB122 and a fragment of the hygromycin B resistance phosphotransferase gene (*HPH*, 1,423bp) cassette in the pBHt2 vector were amplified separately. As illustrated in Supplementary Figure S1, a 5′ fragment of *LtAP1* (1,722bp) amplified with primers 1F/1R was fused with an HY/g (917bp) fragment amplified with primers 2F/2R to generate a construct 5′LtAP1::HY/g; meanwhile, a 3′ fragment of *LtAP1* (1,722bp) amplified with primers 4F/4R was fused with a h/YG (966bp) fragment amplified with primers 3F/3R to produce a construct h/YG::3′LtAP1. As shown in Supplementary Figure S2, the pmCas9 empty vector was digested with *Esp*3I FastDigest (Thermo scientific, United States). A 20bp fragment ahead of NGG in the exon region of *LtAP1* was selected for single-guide RNA (sgRNA) design, and its specificity was tested against the *L. theobromae* genome assembly. The sgRNA sequence was synthesized using primers adapted with sticky ends at the 5′ end (Supplementary Table S1), then inserted into the digested pmCas9 vector by T4 DNA ligase (Thermo Scientific, United States). The inserts in plasmids were then confirmed by sequencing.

Subsequently, two constructs (5′LtAP1::HY/g and h/YG::3′LtAP1) and pmCas9-LtAP1 were mixed and co-transformed into protoplasts prepared from JMB122 using the polyethylene glycol method to create *LtAP1* deletion mutant *ΔLtap1*. The transformants were recovered from a regeneration medium (342gL^−1^ sucrose, 1gL^−1^ yeast extract, 1gL^−1^ casein hydrolysate, and 20gL^−1^ agar) containing 150μgml^−1^ hygromycin B (Roche, Switzerland). The *ΔLtap1* transformants were continuously selected on hygromycin B plates for two generations and verified by PCR.

### Genetic Complementation

The complementation strains were obtained using homologous recombination (Ma et al., 2018). As displayed in Supplementary Figure S1, the full-length ORF of *LtAP1* carrying its native promoter region (1,500bp genomic sequence upstream of the ATG start codon) but without stop codon was amplified with primers 8F/8R from genomic DNA of strain JMB122 and used for genetic complementation of *ΔLtap1*. The amplified PCR fragment was fused with a neomycin resistance gene (*NEO*) cassette under the control of the *Aspergillus nidulans trpC* promoter and terminator, conferring resistance to G418 from plasmid pCETNS. The *LtAP1::NEO* construct was transformed into protoplasts prepared from the mutant *ΔLtap1-8*. The resultant transformants were recovered from the medium amended with 100mgml^−1^ G418 (Sigma, United States) and screened by PCR with primers 5F/5R.

### Virulence Assay

The virulence assay was conducted as previously described (Zhang et al., 2020). The lesion sizes were recorded 5days post-inoculation (dpi). Green bark tissues within 0.5–1.0cm of a wound site were sampled, and immediately placed in liquid nitrogen and stored at −80°C until further analysis. Relative amounts of fungal DNA represented by cycle threshold of *L. theobromae* internal transcribed spacer 1 (LtITS1) were compared to peach-derived elongation factor 1α (*PpEF1α*, reference gene) using the comparative cycle threshold (2^−ΔΔCT^) method (Svetaz et al., 2017). The primers are shown in Supplementary Table S1. Each treatment was tested on 15 peach shoots, and the virulence assay was independently repeated three times.

### Measurement of Superoxide Anion and Hydrogen Peroxide

Absorbance was measured on a spectrophotometer (UV-2450, Shimadzu, Japan). The amount of superoxide anion ($O_{2}^{-}$) and H_2_O_2_ was measured following Zhang et al. (2020). Absorbance at 530nm was recorded to calculate the $O_{2}^{-}$ content expressed in nmol g^−1^ FW. The absorbance levels of H_2_O_2_ (mmolg^−1^ FW) were recorded at 415nm.

### Statistical Analysis

Data were subjected to ANOVA at *p* <0.05. The student’s *t*-test was used to test for significant differences of two-sample treatments at *p* <0.05 or *p* <0.01. Duncan’s multiple range test (*p* <0.05) was used to separate means when there were more than three treatments, and a significant difference was found in the ANOVA.

## Results

### Cloning and Identification of *LtAP1*, a YAP1 Homolog in *Lasiodiplodia theobromae*

The *LtAP1* genomic DNA and cDNA sequences were obtained using primer set FD120/FD121 with the genomic DNA and cDNA of strain JMB122 as templates. The results showed that the *LtAP1* gene contained a 1,945bp coding sequence with a 47bp intron. The *LtAP1* gene (deposited in GenBank with accession number MN933613.1) was predicted to encode a 612 amino acid-long protein that displayed 46 and 43% overall identity with ScYAP1 and MoAP1, respectively. Multiple sequence alignment revealed that AP1s had widely conserved domains: an N-terminal bZIP DNA-binding domain and a nuclear export signal (NES) embedded in a C-terminal cysteine-rich domain (c-CRD; Supplementary Figure S3). Phylogenetic analysis (Supplementary Figure S4) demonstrated that AP1-like proteins were evolutionarily conserved among filamentous fungi and separated from the ScAP1 clade. The LtAP1 amino acid sequence had 56% identity with the AP1 homolog in *Alternaria alternata* (Supplementary Figure S4).

### Generation of *LtAP1* Deletion and Complementation Strains

To investigate the biological function of *LtAP1*, we knocked out the gene. The mutants were confirmed by PCR. The primers 6F/6R and 7F/7R amplified two DNA fragments of 2,747 and 2,714bp, respectively, from genomic DNA of the obtained *ΔLtap1* transformants, while no fragment was obtained from the WT strain, indicating that the *LtAP1* gene was successfully deleted and replaced by the *HYG* gene in the *ΔLtap1* transformants (Supplementary Figure S5). Furthermore, the authenticity of transformants was screened by PCR with primers 5F/5R, and no fragment was amplified, indicating that these transformants were positive deletion mutants. We obtained seven positive transformants, and two (*ΔLtap1-8* and *-10*) were analyzed further.

A 1,178 fragment was amplified from the genomic DNA of complemented strains using primer set 5F/5R, while no fragment was obtained from the knockout transformants, indicating that the WT allele could be re-introduced into the *ΔLtap1* transformants to generate complemented strains (Supplementary Figure S5). We obtained six strains, and strain *ΔLtap1/*AP1 was used in further analyses.

### The Role of *LtAP1* in Mycelial Growth

**Figure 1:**
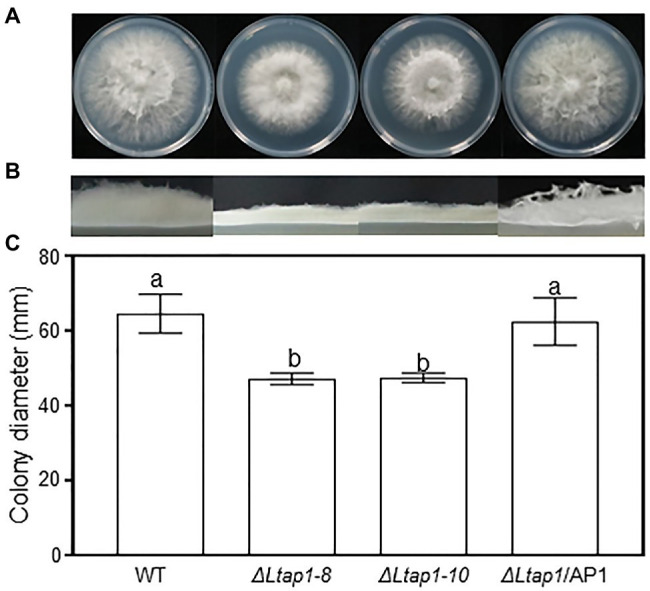
Effect of *LtAP1* deletion on the mycelial growth of *Lasiodiplodia theobromae*. Morphological visualization of fungal colony **(A)**, aerial hyphae growth **(B)**, and diameter quantification **(C)** of the colony growth of WT (wild type), two *ΔLtap1* deletion mutants (*ΔLtap1-8* and *-10*), and the *ΔLtap1/*AP1 complementary strain after 36h at 28°C in darkness. Different letters on top of bars represent a statistically significant differences at *p*<0.05. Bars show mean growth averaged across three biological replicates, and error bars represent standard deviation.

The mycelial growth rate of *ΔLtap1* mutant lines was reduced by 30% compared to the WT strain (Figure 1C). As well, the *ΔLtap1* mutant showed apparent defects in radial growth and aerial hyphal (Figures 1A,B). In contrast, both phenotypes were recovered in the *ΔLtap1/*AP1 strain. The result indicated that the loss of *LtAP1* impaired the vegetative growth of *L. theobromae*.

### Effect of *LtAP1* on Response to Different Exogenous Stresses

**Figure 2:**
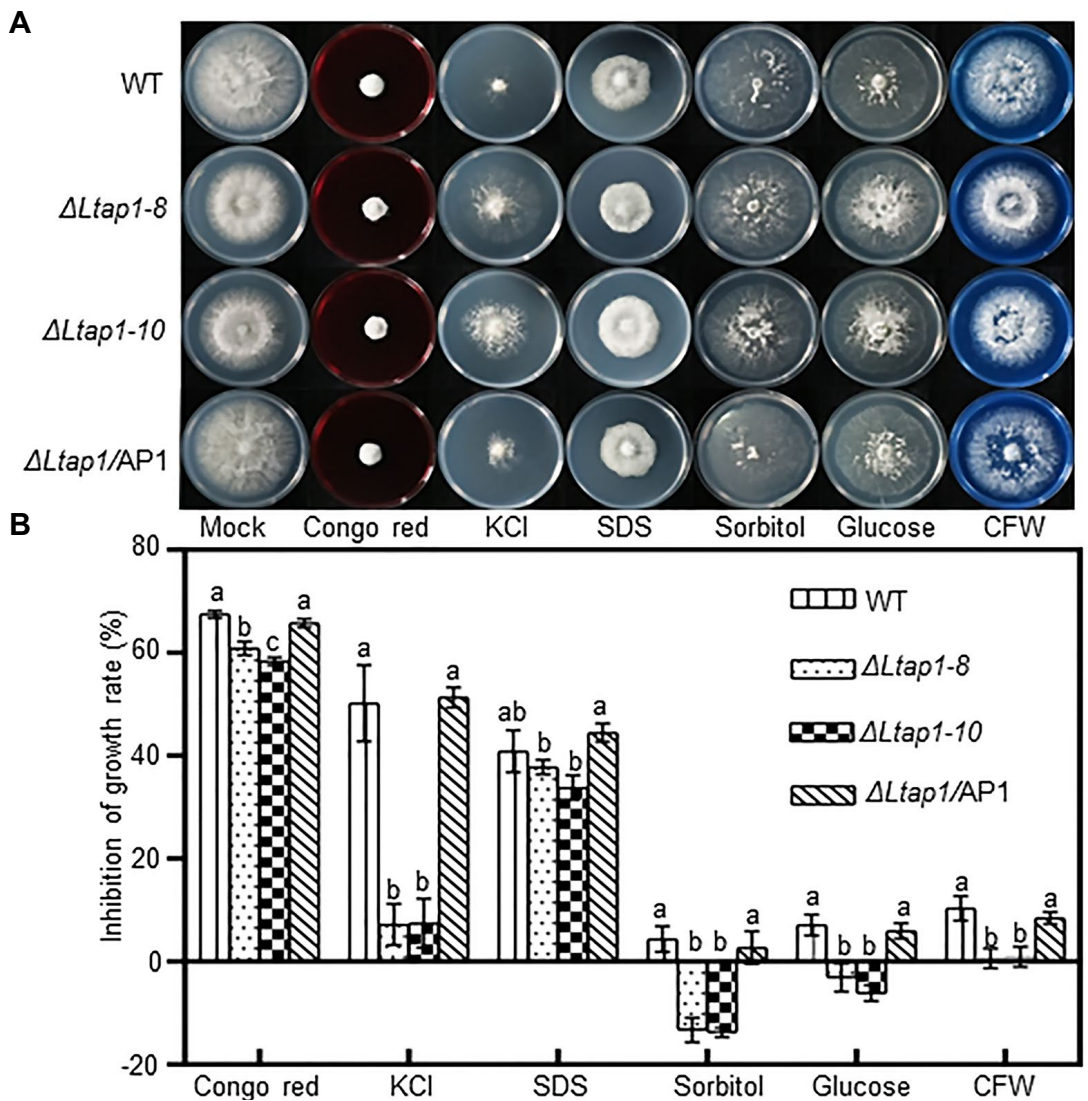
Mycelial growth of the *L. theobromae* WT and mutants in response to stress treatments. **(A)** Cultures of the WT, two *ΔLtap1* deletion mutants and the *ΔLtap1/*AP1 strain, grown on PDA media supplemented with 2.5mgml^−1^ Congo red, 1M KCl, 0.02% SDS, 1M sorbitol, 1M glucose, and 0.05mgml^−1^ calcofluor white (CFW) or water (mock) at indicated concentrations after 36h. **(B)** Percent growth inhibition of WT and mutants on PDA with the inhibitors. Different letters on top of bars represent a statistically significant differences at *p*<0.05. Bars show mean inhibition of growth rate averaged across three biological replicates, and error bars represent standard deviation.

To evaluate whether *LtAP1* can mediate adaptation to exogenous stress, we inoculated mycelial plugs of different genotypes on PDA plates containing cell wall damaging agents (Congo red or CFW), osmotic stress agents (KCl, sorbitol, or glucose), and a cell membrane damaging agent (SDS). After 36h, the mycelial growth in *ΔLtap1* mutants was significantly reduced in Congo red-, KCl-, sorbitol-, glucose-, and CFW-treated plates, while the diameter of *ΔLtap1* mutants was significantly increased in sorbitol- and glucose-amended PDA plates compared to the WT (Figure 2). No significant difference was observed for sensitivity to SDS between *ΔLtap1* mutants and the WT (Figure 2). In all cases, the mycelial morphology and colony diameter of *ΔLtap1/*AP1 under exogenous stress treatments were restored to the WT level (Figure 2).

**Figure 3:**
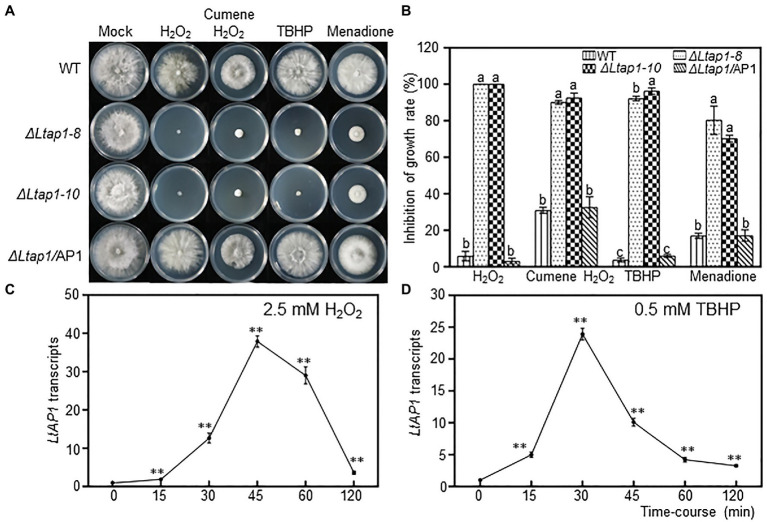
Defects of *LtAP1* on the response of *L. theobromae* to oxidative stress. **(A)** Mycelial growth of WT, two deletion strains and the complementary strain *ΔLtap1/*AP1, cultured on PDA media amended with oxidants 2.5mm H_2_O_2_, 0.68mm cumene H_2_O_2_, 0.5mm *tert*-butyl-hydroperoxide (TBHP), and 0.1mm menadione or water (mock) at the indicated concentrations after 36h. **(B)** Inhibition rate of fungal growth on PDA with oxidants compared with PDA without stress exposure. Different letters on top of bars represent a statistically significant differences at *p*<0.05. Bars show mean inhibition of growth rate averaged across three biological replicates, and error bars represent standard deviation. **(C,D)** Time-course response of *LtAP1* transcripts to H_2_O_2_ or TBHP exposure. Transcript levels were normalized with reference gene *LtTUB* and are displayed relative to the transcript level in samples at time zero (which was therefore set to one). Asterisks indicate the significant difference relative to the initial point (0min) at *p*<0.01. Values are means ± SD of three biological and three technical replicates.

When exposed to H_2_O_2_, cumene H_2_O_2_, TBHP, and menadione treatments, the *ΔLtap1* mutants were much more sensitive to 2.5mm H_2_O_2_, 0.68mm cumene H_2_O_2_, 0.5mm TBHP, and 0.1mm menadione than the WT (Figures 3A,B). The *ΔLtap1* mutants showed a substantial growth reduction compared to the WT at 36hpi, with more than 90% reduction in H_2_O_2_ and TBHP treatments, and approximately 60% reduction in cumene H_2_O_2_ and menadione treatments (Figure 3B). In the *ΔLtap1/*AP1 strain, the stress resistance of strain JMB122 was rescued to the WT level (Figure 3). Further, we tested the transcriptional change of *LtAP1* in WT to exogenous oxidants H_2_O_2_ and TBHP treatment. When compared with untreated mycelia at the initial time point, exposure to 2.5mm H_2_O_2_ increased the transcripts of *LtAP1* quickly at 15min, peaking at 45min, followed by a sharp reduction to the end of monitoring (120min; Figure 3C). Similarly, the expression of *LtAP1* was upregulated rapidly but peaked at 30min under 0.5mm TBHP treatment (Figure 3D).

**Figure 4:**
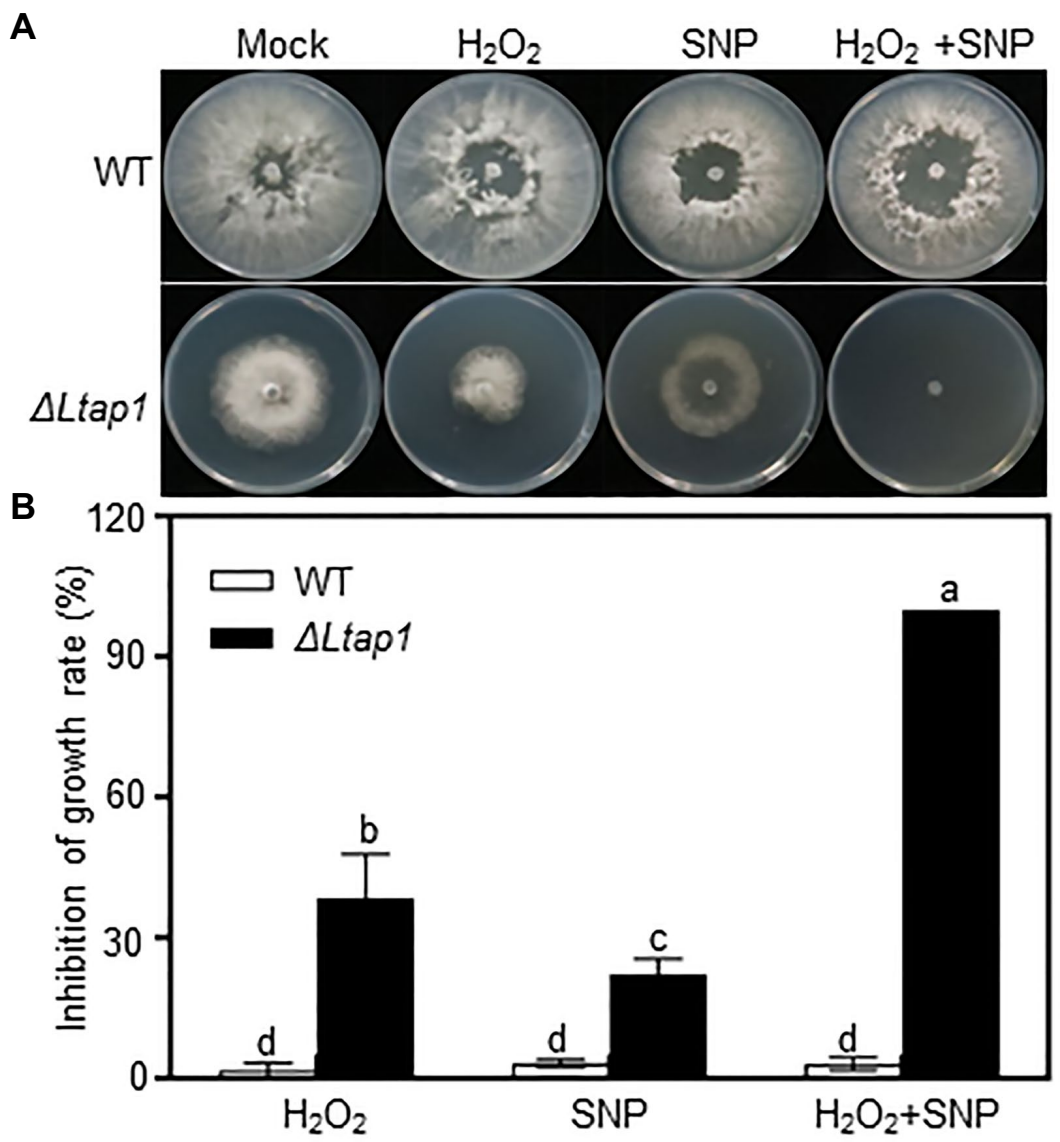
Involvement of *LtAP1* of *L. theobromae* in response to nitro-oxidative stress. **(A)** Fungal growth of WT and the deletion mutant strain *ΔLtap1* cultured on PDA stressed with 1mm H_2_O_2_ and/or 5mm Sodium nitroferricyanide dihydrate (SNP), or water (mock) at the indicated concentration after 36h. **(B)** Inhibition rate of fungal growth on PDA with stress treatment in relation to the mock. Values are means ± SD of three biological replicates. Different letters on top of paired bars represent a statistically significant difference at *p*<0.05.

Additionally, we also tested the involvement of *LtAP1* in nitrosative stress tolerance. The mycelial growth of the *ΔLtap1* strain was significantly reduced in the SNP treatment compared with the WT (Figure 4). Moreover, the growth inhibition of the *ΔLtap1* mutant was higher in the treatments with SNP and H_2_O_2_ together than in the single treatments with SNP or H_2_O_2_ (Figure 4).

### Pathogenicity of the *LtAP1* Mutant Strain on Peach Shoots

**Figure 5:**
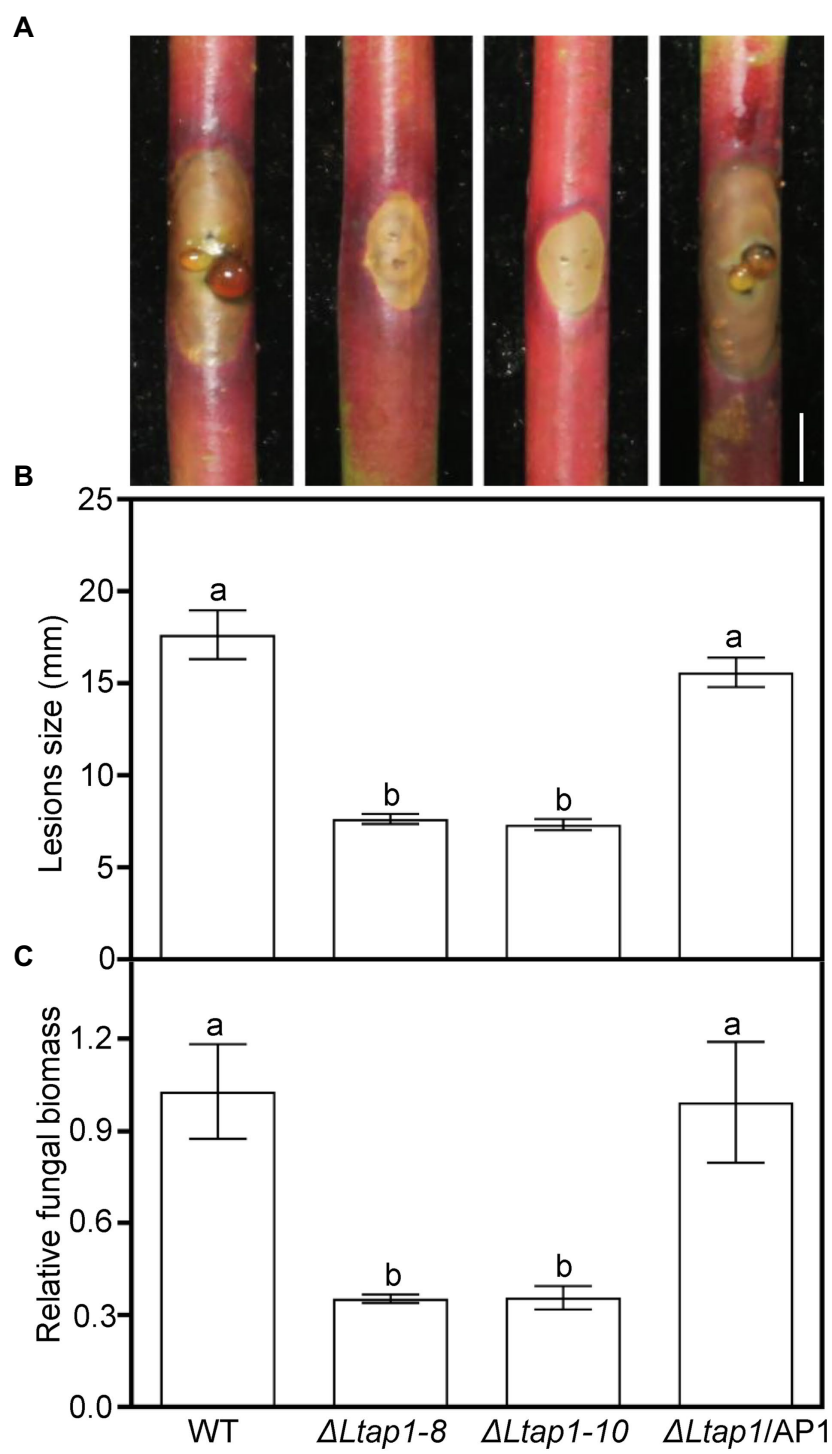
Virulence test of the *LtAP1* mutants into peach shoots. **(A)** Peach gummosis progression in the detached shoots inoculated with different genotypes of *L. theobromae* (WT, two deletion mutants, and the complementary strain *ΔLtap1*/AP1) at 5 dpi. Bar represents 5mm. **(B)** Quantification of lesion size on inoculated peach shoots. **(C)** Quantitative real-time PCR (qRT-PCR) analysis of *L. theobromae* amounts in the infected peach shoots. In panels, different letters on top of bars indicate statistically significant differences at *p*<0.05.

Pathogenicity assays on detached current-year peach shoots revealed that the *ΔLtap1* strains caused small brown necrotic lesions and invisible gum release at the site of fungal inoculation, when compared with the WT at 5 dpi, the last observation time (Figure 5A). The *ΔLtap1*/AP1 induced necrotic lesions at a rate and magnitude comparable to the WT (Figure 5A). Quantitative analysis demonstrated that the size of lesions induced by the *ΔLtap1* mutants was about 43% of that caused by the WT (Figure 5B). Furthermore, the relative fungal biomass (as revealed by qPCR) in infected peach shoots of the *ΔLtap1* mutants was significantly less than that of the WT (Figure 5C). The lesion sizes and fungal biomass in the *ΔLtap1*/AP1 strain-inoculated peach shoots were rescued to WT levels (Figures 5B,C).

### Effect of *LtAP1* Deficiency on ROS Accumulation in Infected Peach Shoots

**Figure 6:**
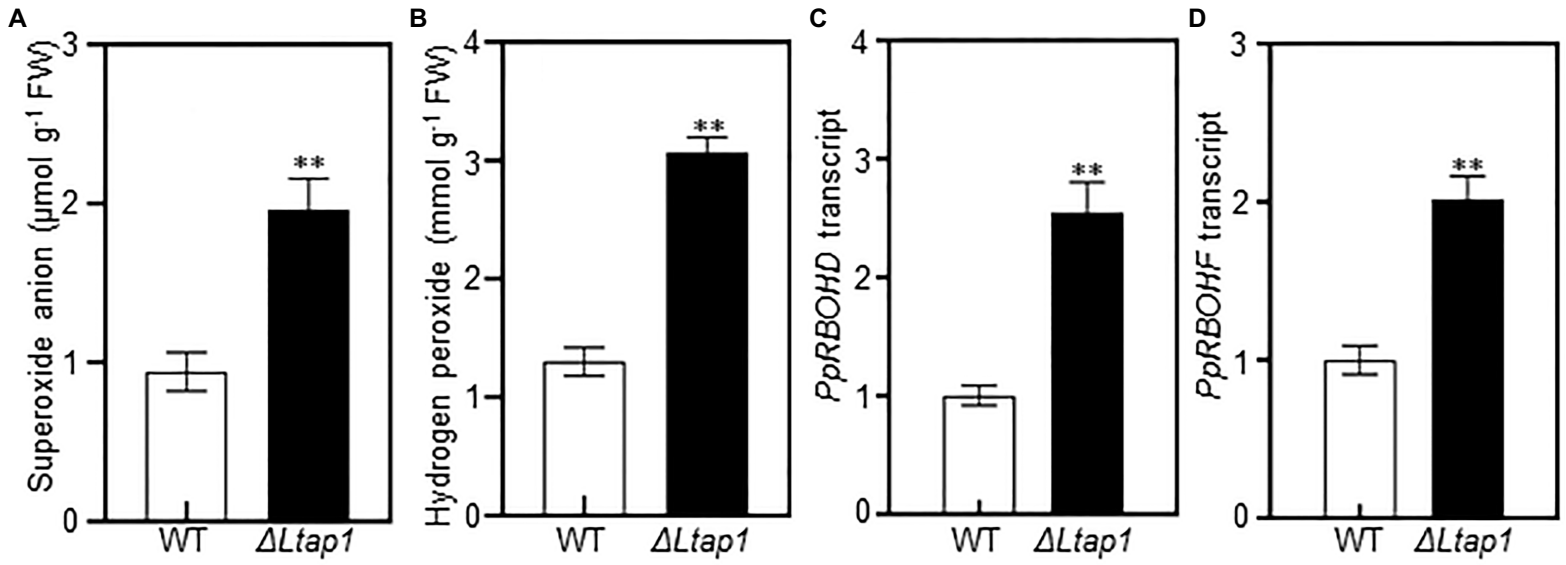
Effect of *LtAP1* deficiency on ROS generation and its related genes transcripts in infected peach shoots. **(A,B)**: Accumulation of superoxide anion and hydrogen peroxide in peach shoots inoculated with *L. theobromae* WT or *ΔLtap1* mutant at 5 dpi. **(C, D)**: Transcript abundance of ROS production-related genes *PpRBOHD* and *PpRBOHF* in infected peach shoots at 5 dpi. Relative transcript levels of genes compared with that of the control using reference gene *PpTEF2* for normalization. All data are means ± SD of three biological replicates. Asterisks indicate the significant difference between two genotypes for the same parameter comparison at *p*<0.01.

To test the involvement of *LtAP1* in scavenging ROS, $O_{2}^{-}$ and H_2_O_2_ contents were measured in peach shoots infected by *ΔLtap1* mutant or WT at 5 dpi. Both $O_{2}^{-}$ and H_2_O_2_ contents were significantly increased, respectively, with 1.1- and 2.6-fold higher levels in the *ΔLtap1* mutant-inoculated shoots than the controls (Figures 6A,B). Furthermore, we tested whether *LtAP1* was involved in the regulation of ROS production during infection. The transcripts of core ROS production-related genes, *PpRBOHs*, were examined. Our data showed that the expression levels of both *PpRBOHD* and *PpRBOHF* were significantly higher in shoots inoculated with *ΔLtap1* mutant than the WT (Figures 6C,D).

### Effects of Prevention of ROS Generation on Pathogenicity of the *LtAP1* Mutants

**Figure 7:**
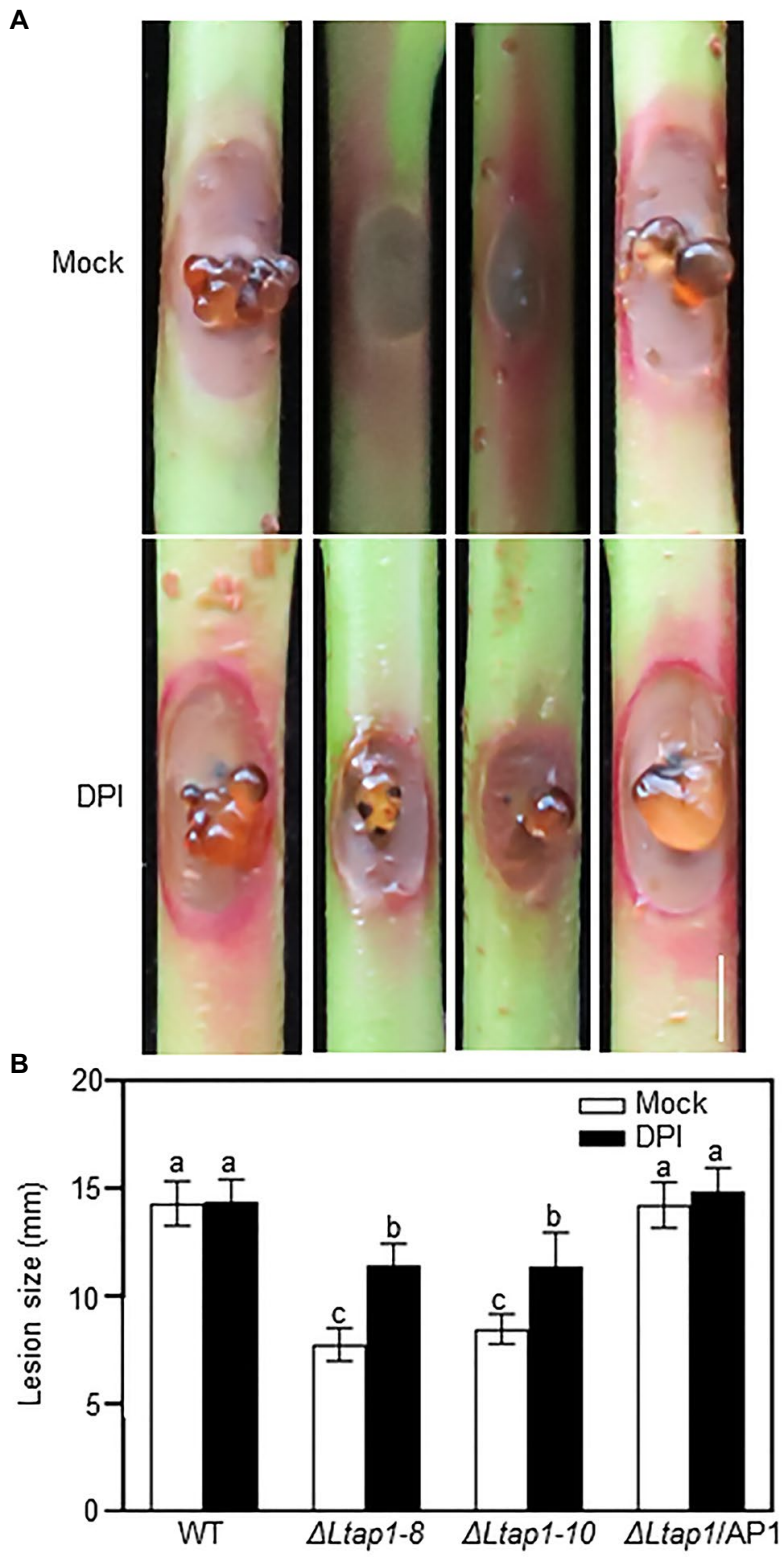
Impact of diphenylene iodonium (DPI) on pathogenicity of *LtAP1* mutants. **(A)**: Symptom of different genotypes of *L. theobroame*-inoculated peach shoots was treated with 0.4μm DPI [dissolved in dimethyl sulfoxide (DMSO) and then diluted with water; an NADPH oxidase inhibitor] or not (mock, 0.04% DMSO) at 5 dpi. Bar represents 5mm. **(B)** Quantification of lesion size in infected shoots. All data are means ± SD of three biological replicates. Different letters indicate the significant difference at *p*<0.05.

To elucidate the role of LtAP1-modulating oxidative stress tolerance in fungal pathogenicity, an NADPH oxidase inhibitor, DPI, was used in virulence testing of the *ΔLtap1* mutants. We observed that the *ΔLtap1* mutants induced much larger brown necrotic lesions and more visible gum release in the DPI-treated shoots than the mock-treated ones at 5 dpi (Figures 7A,B). Moreover, in the DPI-treated shoots, the size of lesions induced by the *ΔLtap1* mutants was only about 79% of that caused by the WT (Figure 7B).

### Role of *LtAP1* in the Expression of ROS Detoxification-Related Genes in *L. theobromae*

**Figure 8:**
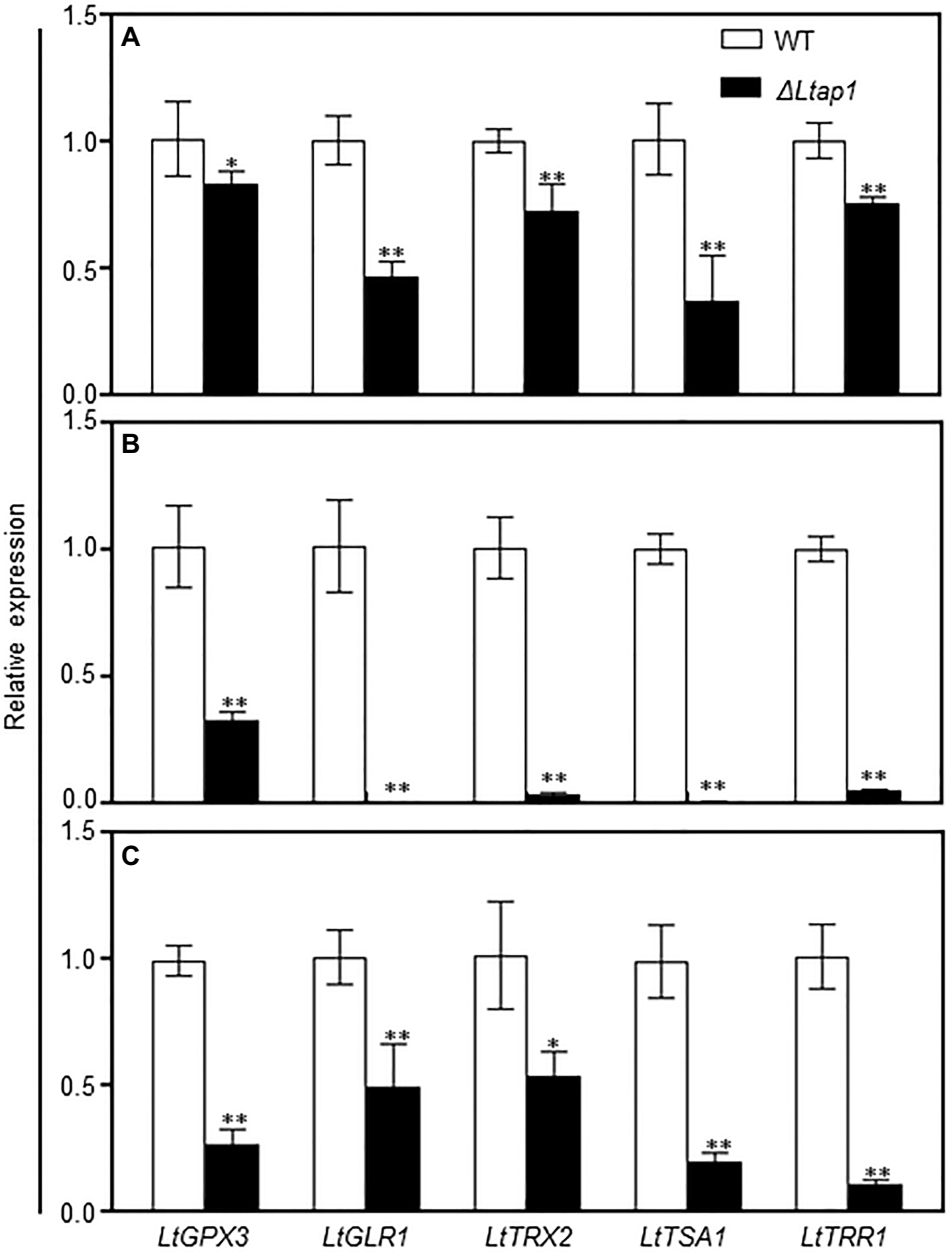
qRT-PCR analysis of the glutaredoxin and thioredoxin systems genes in the WT, *LtAP1* deletion mutant under H_2_O_2_ treatment and in infected peach shoots. Mycelial samples of WT and the deletion mutant *ΔLtap1* treated with water **(A)** and 2.5mm H_2_O_2_ **(B)** were collected after 1h culture at 28°C in darkness. **(C)** RNA samples were collected from the border of *L. theobromae*-colonized peach shoots at 5 dpi. The transcript levels were normalized with *LtTUB* and are displayed in relation to the transcript levels in the corresponding WT samples (which was therefore set to one). The values are means ± SD of three biological replicates. Asterisks indicate significant differences for genes between two genotypes, with ^*^p<0.01 and ^**^p<0.05.

To identify genes regulated by LtAP1, the transcripts of genes related to antioxidants (glutaredoxin and thioredoxin) were analyzed in the WT and the *ΔLtap1* mutant exposed to 2.5mm H_2_O_2_ or distilled water for 1h. Relative transcripts of the core genes of both the glutaredoxin system (*LtGPX3* and *LtGLR1*) and the thioredoxin system (*LtTRX2*, *LtTSA1*, and *LtTRR1*) were significantly lower in the *ΔLtap1* mutant in the absence of H_2_O_2_ (Figure 8A). Under H_2_O_2_ treatment, the expression of all tested genes was consistently and significantly further decreased to 68 to 100% in the *ΔLtap1* mutant, as compared to the WT (Figure 8B).

To further elucidate the possible mechanism behind the impairment of oxidative stress response and pathogenicity in the *ΔLtap1* mutant, transcript levels of genes in the glutaredoxin and thioredoxin systems were assayed for *ΔLtap1* or WT infected tissues. Indeed, the inactivation of *LtAP1* led to significant reductions of all tested genes expression, ranging from 49 to 90% in the shoots inoculated with *ΔLtap1* relative to the WT at 5 dpi (Figure 8C).

### Effect of *LtAP1* Deletion on Plant Defense Response

**Figure 9:**
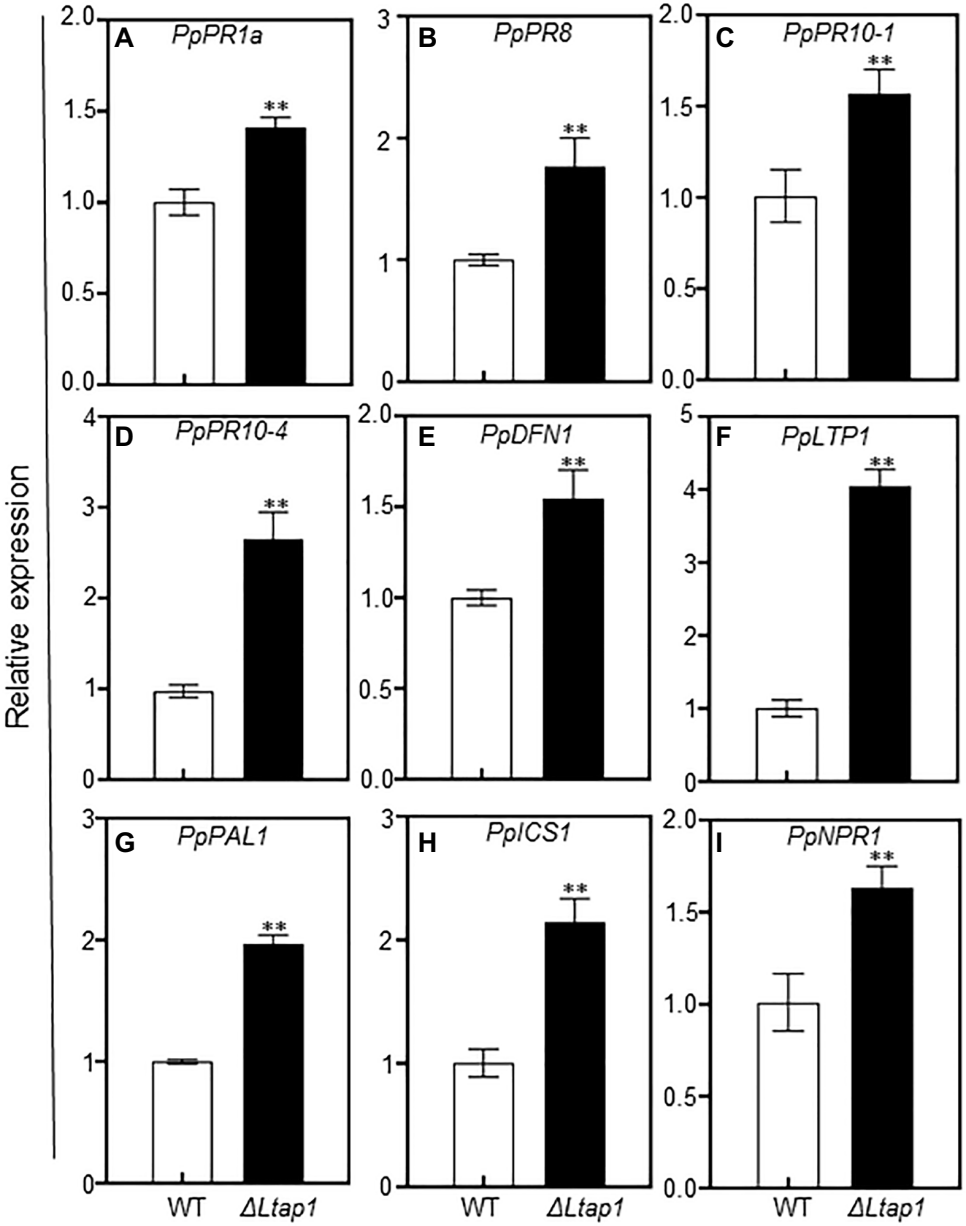
Impact of *LtAP1* deficiency on the transcripts of defense-responsive genes in infected peach shoots. RNA samples were collected from peach shoots inoculated with *L. theobromae* WT or *ΔLtap1* mutant at 5 dpi. **(A–F)**: The relative expression of pathogenesis-related (*PR*) genes, including *PpPR1a*, *PpPR8*, *PpPR10-1*, *PpPR10-4*, *PpDFN1*, and *PpLTP1*. **(G–I)**: Expression pattern of SA biosynthetic (*PpPAL1* and *PpICS1*) and signaling (*PpNPR1*) genes. Relative transcript levels of genes compared with that of the control using reference gene *PpTEF2* for normalization. Values are means ± SD of three biological replicates. Asterisks indicate a significant difference between two genotypes for genes at *p*<0.01.

ROS often act as signaling molecules to activate defense-related genes, such as pathogenesis-related (*PR*) genes, to enhance plant defense response (Camejo et al., 2016). To further assess whether *PR* genes were activated by the *ΔLtap1* mutant inoculation, transcripts of several *PR* genes, including *PpPR1a*, *PpPR8*, *PpPR10-1*, *PpPR10-4*, *PpDFN1* (Defensin 1, PR12 family), and *PpLTP1* (Lipid-transfer protein 1, PR14 family), were examined at 5 dpi in the peach shoots inoculated with the *ΔLtap1* mutant or WT. The transcripts of all tested *PR* genes were significantly higher in shoots inoculated with *ΔLtap1* than WT (Figures 9A–F). Notably, the transcripts of *PpPR10-4* and *PpLTP1* were 2.7- and 4.0-fold higher, respectively, in tissues inoculated with the *ΔLtap1* than the control (Figures 9D,F). In addition, the transcripts of plant defense-related gene *PpPAL1* (Phenylalanine ammonia lyase 1) were also significantly induced and were 2.0-fold higher in the *ΔLtap1*-inoculated shoots than those with the control (Figure 9G). The transcripts of *PpICS1* (isochorismate synthase 1) and *PpNPR1* (nonexpressor of pathogenesis-related gene 1), which were required for SA biosynthesis and signal transduction, were also significantly upregulated in the peach shoots inoculated with the *ΔLtap1* mutant than the WT (Figures 9H–I).

## Discussion

The peach gummosis pathogen, *L. theobromae*, is a destructive threat to peach harvests (Wang et al., 2011), and infection events at the molecular level need deeper investigation. In a previous study, *L. theobromae* infection caused an oxidative burst in peach shoots and promoted expression of *LtAP1* and other genes associated with the ROS scavenging system (Zhang et al., 2020). Subsequently, we attempted to uncover how plant infection is regulated by an oxidative stress regulator, YAP1, in *L. theobromae*.

Eukaryotic microorganisms have stress-protective functions against a variety of adverse conditions. We first compared the growth performance of the *ΔLtap1* mutant and the WT grown on media supplemented with different exogenous chemicals to mimic environmental stresses. Our results suggest that in *L. theobromae*, *LtAP1* was involved in response to various stresses. Deletion of *LtAP1* led to decreased sensitivity to osmotic and cell wall inhibitors, indicating that *LtAP1* negatively regulated the sensitivity to osmotic pressure and the maintenance of cell wall integrity in *L. theobromae* (Figure 2). Likewise, in *C. gloeosporioides*, *ΔCgap1* mutants had higher resistance to sorbitol than the WT (Li et al., 2017). However, in *F. graminearum*, the *Fgap1*-deficiency mutant exhibited increased sensitivity to sorbitol or NaCl-induced stresses (Montibus et al., 2013). In addition, our oxidative stress tests indicated that *ΔLtap1* mutants were hypersensitive to H_2_O_2_, cumene H_2_O_2_, and TBHP, as well as menadione (Figures 3A,B). These results suggest that *LtAP1* plays a vital role in the regulation of fungal response to oxidative stress. This is consistent with studies on *M. oryzae* (Guo et al., 2011), *F. graminearum* (Montibus et al., 2013), *A. alternata* (Lin et al., 2009), and *C. gloeosporioides* (Sun et al., 2016), where the mycelial growth of the respective mutant was severely reduced by oxidative stress compared to their respective WT. Moreover, the expression levels of *LtAP1* were significantly upregulated under the oxidant treatments (Figures 3C,D). A similar finding was observed in *C. gloeosporioides* (Sun et al., 2016) and *Monilinia fructicola* (Yu et al., 2017). The results suggest that the fungal YAP1s transcription factors are highly conserved for oxidative stress response in different species.

Interestingly, the *ΔLtap1* mutant showed a significant reduction of mycelial growth with the SNP treatment. The growth suppression of the *ΔLtap1* mutant after treatment with SNP plus H_2_O_2_ was higher than of either SNP or H_2_O_2_ alone (Figure 4), which showed an additive effect between SNP and H_2_O_2_. ROS can react with nitric oxide and generate toxic reactive nitrogen species (RNS), such as peroxynitrite (Marroquin-Guzman et al., 2017). Hence, *LtAP1* is likely an essential player in oxidative and nitrosative stress adaptation.

Pathogenicity tests revealed that the *ΔLtap1* mutant induced smaller necrotic lesions, less gum release, and decreased pathogen biomass than WT (Figure 5), suggesting that *LtAP1* is essential for growth and virulence of the necrotrophic fungus *L. theobromae* on peach shoots. Similarly, in the biotrophic *U. maydis* and necrotrophic *A. alternata*, deletion of *AP1* failed to incite necrotic lesions (Molina and Kahmann, 2007; Lin et al., 2009). In the hemibiotrophic pathogen *M. oryzae*, *Moap1* is essential to the growth of invasive hyphae for successful infection (Guo et al., 2011). In hemibiotrophic *C. gloeosporioides*, *ΔCgap1* mutant showed severely attenuated virulence on poplar leaves (Sun et al., 2016) and could not induce lesions on mango fruits (Li et al., 2017). However, in necrotrophic *B. cinerea* (Temme and Tudzynski, 2009) and *F. graminearum* (Montibus et al., 2013), the deletion of *AP1* did not show noticeable effects on pathogenicity, indicating that YAP1 homologs are not necessary for virulence in all pathogenic fungi studied. This might be because fungal virulence associated with YAP1 differs in the types of associations established between specific fungi and plant hosts. A better and deeper understanding of the mechanisms of pathogen virulence associated with YAP1 homologs is needed.

It is well known that a major mechanism of plant defense is the production of ROS against pathogens attack. Therefore, fungal pathogens need robust strategies for ROS scavenging, which involves YAP1 homologs (Segal and Wilson, 2018). In the *L. theobromae*-infected peach shoots, the expression of *LtAP1*, the glutaredoxin system genes (*LtGPX3* and *LtGLR1*), and the thioredoxin system genes (*LtTRX2*, *LtTSA1*, and *LtTRR1*) was markedly upregulated, which was perhaps to scavenge ROS derived from the host (Zhang et al., 2020). In this study, we found higher ROS accumulation at the inoculation site with the mutant than with the WT (Figures 6A,B). Moreover, the *ΔLtap1* mutants were hypersensitive to exogenous oxidative stress (Figures 3A,B). This suggested that LtAP1-modulating oxidative stress tolerance might play a crucial role in fungal pathogenicity. To further investigate the link between LtAP1 modulation of oxidative stress tolerance and fungal pathogenicity, we used an NADPH oxidase inhibitor, DPI, to prevent ROS generation. The results clearly showed that the DPI treatment increased necrotic lesion size and enhanced gum release in the shoots inoculated with the *ΔLtap1* mutants as compared to the mock control, suggesting that the pathogenicity of *ΔLtap1* mutants was partially restored (Figures 7A,B). Overall, LtAP1 modulation of oxidative stress tolerance, at least in part, contributed to the pathogenicity of *L. theobromae*. Similarly, the *ΔAaAP1* mutant of necrotrophic *A. alternata* was hypersensitive to oxidants, and its pathogenicity was rescued by the NADPH oxidase inhibitor treatment (Lin et al., 2009). In biotrophic *U. maydis*, H_2_O_2_ was markedly accumulated at sites inoculated with the *Umap1* mutant, and inhibition of the plant NADPH oxidase decreased ROS accumulation and restored the virulence of the mutant, suggesting that *Umap1* acts in neutralizing the ROS generated by the maize NADPH oxidase (Molina and Kahmann, 2007).

In filamentous fungi, YAP1 homologs are major regulators of the antioxidant response, but YAP1 homologs involve a wide array of processes by regulating genes involved in ROS scavenging (Mendoza-Martínez et al., 2020). The expression of such genes, such as the core glutaredoxin system genes (*LtGPX3* and *LtGLR1*) and thioredoxin system members (*LtTRX2*, *LtTSA1*, and *LtTRR1*), was dramatically downregulated in the *ΔLtap1* mutant treated with water or H_2_O_2_ (Figures 8A,B), indicating that *LtAP1* acts as a major regulator in the antioxidant system. Similarly, the transcription factor *AaAP1* could activate glutaredoxin (*AaGPX3* and *AaGLR1*) and thioredoxin systems (*AaTSA1* and *AaTRR1*) to cope with oxidative stress (Yang et al., 2016; Ma et al., 2018). The thioredoxin *MoTrx2* was found to be a target of the transcription factor MoAP1 in *M. oryzae*, and *ΔMotrx2* mutant displayed higher ROS levels and lower POD and laccase activities (Wang et al., 2017). However, in *F. graminearum*, the expression of three CAT- and two Cu/ZnSOD-encoding genes was downregulated in the *Fgap1* mutant (Montibus et al., 2013). Likewise, EfAP1 in *Epichloe festucae* was required for expression levels of the *CAT* gene (Cartwright and Scott, 2013). As a whole, it suggests that YAP1 homologs could regulate/target different antioxidant system-related genes to overcome oxidative stress in different fungi.

During infection, the transcript levels of genes in glutaredoxin and thioredoxin systems were significantly downregulated in the *ΔLtap1* mutant (Figure 8C). Concomitantly, the contents of $O_{2}^{-}$ and H_2_O_2_ and transcripts of *PpRBOHs* were markedly higher in shoots inoculated with the *ΔLtap1* mutant, demonstrating a reduced ability of the *ΔLtap1* mutant to scavenge overproduced ROS during the interaction (Figure 6). It is speculated that LtAP1 is likely to modulate glutaredoxin and thioredoxin systems to scavenge host-derived ROS. Similarly, *AaAP1* could also modulate glutaredoxin and thioredoxin systems to cope with oxidative stress (Lin et al., 2009; Yang et al., 2016; Ma et al., 2018).

ROS serving as the primary signaling molecule during pathogens attack can activate an array of defense responses, such as induction of defense-related genes (Qi et al., 2017; Segal and Wilson, 2018). We observed significantly higher expression levels of *PR* genes, such as *PpPR1a*, *PpPR8*, *PpPR10-1*, *PpPR10-4*, *PpDFN1*, and *PpLTP1*, in the *ΔLtap1* mutant-infected shoots in comparison with the WT treated (Figures 9A–F). Furthermore, the transcripts of SA biosynthesis and signaling-related genes (*PpPAL1*, *PpICS1*, and *PpNPR1*) were also significantly upregulated after *ΔLtap1* mutant inoculation than the WT (Figures 9G–I). The accumulation of PR proteins and SA-mediated plant defense response might assist in limiting disease development, which was reflected by the reduced lesion size, gum release, and fungal biomass at inoculation sites with the *ΔLtap1* mutant (Figure 5). Similarly, the thioredoxin *MoTrx2* regulated by MoAP1 played an essential role in the ROS scavenging during host invasion and in the suppression of the rice defense response, in which the transcript levels of plant defense genes were markedly higher in rice cells infected with the *ΔMotrx2* mutant than the control (Wang et al., 2017). The rice cells inoculated with the *ΔModes1* mutant exhibited strong defense responses accompanied by the accumulation of ROS and *PR* genes transcript in neighboring tissues, indicating that *DES1* is required to suppress the host basal defenses (Guo et al., 2011). Taken together, we propose that the restricted expansion of the *ΔLtap1* mutant in peach shoots is partly caused by the defect in active suppression of peach defense response.

In summary, we cloned and characterized the *LtAP1* gene, which encodes a homolog of yeast YAP1. Our experiments demonstrated that LtAP1 was valuable for mycelial growth, stress response, and pathogenicity. We found that LtAP1 was a key regulator of oxidative stress response, acting in activating fungal glutaredoxin and thioredoxin systems, and suppressing plant defense responses during infection. The prevention of ROS production could partially restore pathogenicity of *ΔLtap1* mutant. *LtAP1* plays a central role in adjusting ROS homeostasis between fungal pathogen and plant host and is necessary for full virulence of *L. theobromae*. This study advances our understanding of the link between oxidative stress response, ROS detoxification, and virulence in *L. theobromae*. Given the critical roles of LtAP1 in *L. theobromae*-induced peach gummosis, it would be urgent to identify its potential targets in the downstream network, which would be helpful for future disease management.

## Data Availability Statement

The datasets presented in this study can be found in online repositories. The names of the repository/repositories and accession number(s) can be found at: https://www.ncbi.nlm.nih.gov/genbank/, MN933613.1.

## Author Contributions

HZ, DZ, GL, and JL designed the experiments. HZ performed all the experiments with occasional help from WS, DZ, and XS. HZ, WS, and DZ analyzed the data. FW provided the analytical tools. HZ, JL, and TH wrote the manuscript. All authors read and approved the final manuscript.

## Funding

This work was financially supported by the China’s National Key Research and Development Program (grant no. 2018YFD1000300) to JL, the Natural Science Foundation of China (grant no. 31471840) and the China Agriculture Research System of MOF and MARA (grant no. CARS-30) to GL, and the Central Public-interest Scientific Institution Basal Research Fund for Chinese Academy of Tropical Agricultural Sciences (grant nos. 1630092021006 and 16300920210010) to HZ.

## Conflict of Interest

The authors declare that the research was conducted in the absence of any commercial or financial relationships that could be construed as a potential conflict of interest.

## Publisher’s Note

All claims expressed in this article are solely those of the authors and do not necessarily represent those of their affiliated organizations, or those of the publisher, the editors and the reviewers. Any product that may be evaluated in this article, or claim that may be made by its manufacturer, is not guaranteed or endorsed by the publisher.
