# Supplementary material for: The bZIP Transcription Factor LtAP1 Modulates Oxidative Stress Tolerance and Virulence in the Peach Gummosis Fungus Lasiodiplodia theobromae
Source: Front Microbiol. 2021 Sep 23;12:741842. doi: 10.3389/fmicb.2021.741842 (PMC8495313; doi:10.3389/fmicb.2021.741842)
Supplement: Supplementary file 1 [file Table_1.DOCX]

**Table S1 Primers used in this study**

| **Primer** | **Primer Sequence (5’-3’)** | **Purpose** |
| --- | --- | --- |
| FD120 | ATGGCGGGCACCACCAACAATGAC | Amplification of *LtAP1* cDNA |
| FD121 | TTAGGCCTTGTCTGGTAGCCTCTTGA |  |
| 1F | TCACCAGACTTCCACTTCGC | *LtAP1* deletion vector construction |
| 1R | TCAATATCATCTTCTGTCGATATACGGGCAGAAGCGTTCC |  |
| 2F | TCGACAGAAGATGATATTGAAGGAG |  |
| 2R | AGCATCAGCTCATCGAGAGCCT |  |
| 3F | AGGGCGAAGAATCTCGTGCTTT |  |
| 3R | GTTAAGTGGATCCCGGTCGGCATCT |  |
| 4F | CGACCGGGATCCACTTAACTCGCTTCCACACTCGAACT |  |
| 4R | CTCGTACCGCAGACACATGG |  |
| 5F | GGAAGGCGAGGATAAGACGG | RT-PCR validation of *LtAP1* |
| 5R | GCCGGACTTTGGTTGTTGTC |  |
| 6F | GGCTCCTTCTTCGCTTGG | RT-PCR validation of *HYG* gene integrity |
| 6R | TATTGACCGATTCCTTGCGG |  |
| 7F | GAAGTGCTTGACATTGGGGA |  |
| 7R | AATCCTCGGACCGCATTCTC |  |
| 8F | GCTCAACTTCAAGGTTTGCATTCG | Complemented vector construction of *LtAP1* |
| 8R | AATATCATCTTCTGGGCCTTGTCTGGTAGCCTCT |  |
| 9F | ACCAGACAAGGCCCAGAAGATGATATTGAAGGAGCACTTTTTGG |  |
| 9R | AAAGAAGGATTACCTCTAAACAAGTGTACCTGT |  |
| FD122 | AATCGGTGCTGCTTTCTGG | *LtTUBULIN* (HQ660474)  qRT-PCR |
| FD123 | TTGTTGGACGCCTCGTTG |  |
| FD124 | TCTCTTGGTTCTGGCATCGA | *LtITS* (HQ660464)  qRT-PCR |
| FD125 | TAATGACGCTCGAACAGGCA |  |
| FD126 | TTTCCCTCAAGATGGTGCCG | *LtAP1* (MN933613)  qRT-PCR |
| FD127 | TCACCAGTCTCTGTTTCGCC |  |
| FD128 | TCCAGTTCTTGATTGCCACA | *PpEF1α* (XM_007222067)  qRT-PCR |
| FD129 | CCATACCTGCATCTCCGTTC |  |
| FD130 | CTTCGTCATCCTCGGCTTCC | *LtGPX3* (MN933616)  qRT-PCR |
| FD131 | CGGGCTTCTCGCTCTTCAT |  |
| FD132 | TCGGTTCTATCGGTTTGTCC | *LtGLR1* (MN933615)  qRT-PCR |
| FD133 | TCGCCCAGAATGTGAAGG |  |
| FD134 | CTCGTCGTCCTCGACTGCT | *LtTRX2* (MN933625)  qRT-PCR |
| FD135 | ACCTTCTCACCGCCCTTG |  |
| FD136 | GCGATGCCGTCCAGCAGTT | *LtTSA1* (MN933626)  qRT-PCR |
| FD137 | TGTTGATGCCGCCGAGACC |  |
| FD138 | ATGGGCGACGATGATACCC | *LtTRR1* (MN933624)  qRT-PCR |
| FD139 | GTTGCGGAAGATTGGAGC |  |
| FD140 | TCTGCAGTACAAGAATAAGGCA | *PpRBOHD* (XM_007225300)  qRT-PCR |
| FD141 | GCCCAATTTGGTTTTGTTTCTG |  |
| FD142 | TTCAACATGGCTCTGGTGCT | *PpRBOHF* (XM_007210336)  qRT-PCR |
| FD143 | TCACAGGCAAGATGGTTCCC |  |
| FD144 | GGTAGGTGTTGCTCCCTTGAC | *PpPR1a* (JF694923)  qRT-PCR |
| FD145 | CGGTGCTCTTCGCTATGTTTT |  |
| FD146 | TCCAAGTCCAGCAGTTTGTG | *PpICS1* (XM_007209022)  qRT-PCR |
| FD147 | TCTCTCCTCCTCCAAACCAA |  |
| FD148 | CGGCTTACTGCGATCCTAAG | *PpNPR1* (XM_007202810)  qRT-PCR |
| FD149 | TGCACGAGCTCCTTTAGTCA |  |
| FD150 | TAAAGAGCACCAGCAACTACCA | *PpPR10-4* (XM_007223565)  qRT-PCR |
| FD151 | TAAAATGAAAGGCCACACACAC |  |
| FD152 | TGGGGGCCCTAGAGACAGTA | *PpPR8* (XM_007211604)  qRT-PCR |
| FD153 | TTTGTCTTGGCAGGATTGCG |  |
| FD154 | TTGATCGAAGGAGATGCTTTGGG | *PpPR10-1* (EU117120)  qRT-PCR |
| FD155 | ATTTAGTTGTAGGCATCGGGGTG |  |
| FD156 | GGCATTAGGAACGTCAACAACT | *PpLTP1* (XM_007206097)  qRT-PCR |
| FD157 | GTCCCATATCAAGCTCACTTCAC |  |
| FD158 | CAACTGCCTTCGTCTTCTTTCT | *PpDFN1* (AY078426)  qRT-PCR |
| FD159 | AATGTGCATGCAGGAGTATCAC |  |
| FD160 | TTTTGAGGAAGAGCTGAAGGTG | *PpPAL1* (XM_007208370)  qRT-PCR |
| FD161 | CTTTCCCTCGCAGATAGCTGA |  |
