# Supplementary material for: The bZIP Transcription Factor LtAP1 Modulates Oxidative Stress Tolerance and Virulence in the Peach Gummosis Fungus Lasiodiplodia theobromae
Source: Front Microbiol. 2021 Sep 23;12:741842. doi: 10.3389/fmicb.2021.741842 (PMC8495313; doi:10.3389/fmicb.2021.741842)
Supplement: Supplementary file 2 [file Presentation_1.PPTX]

## Slide 1
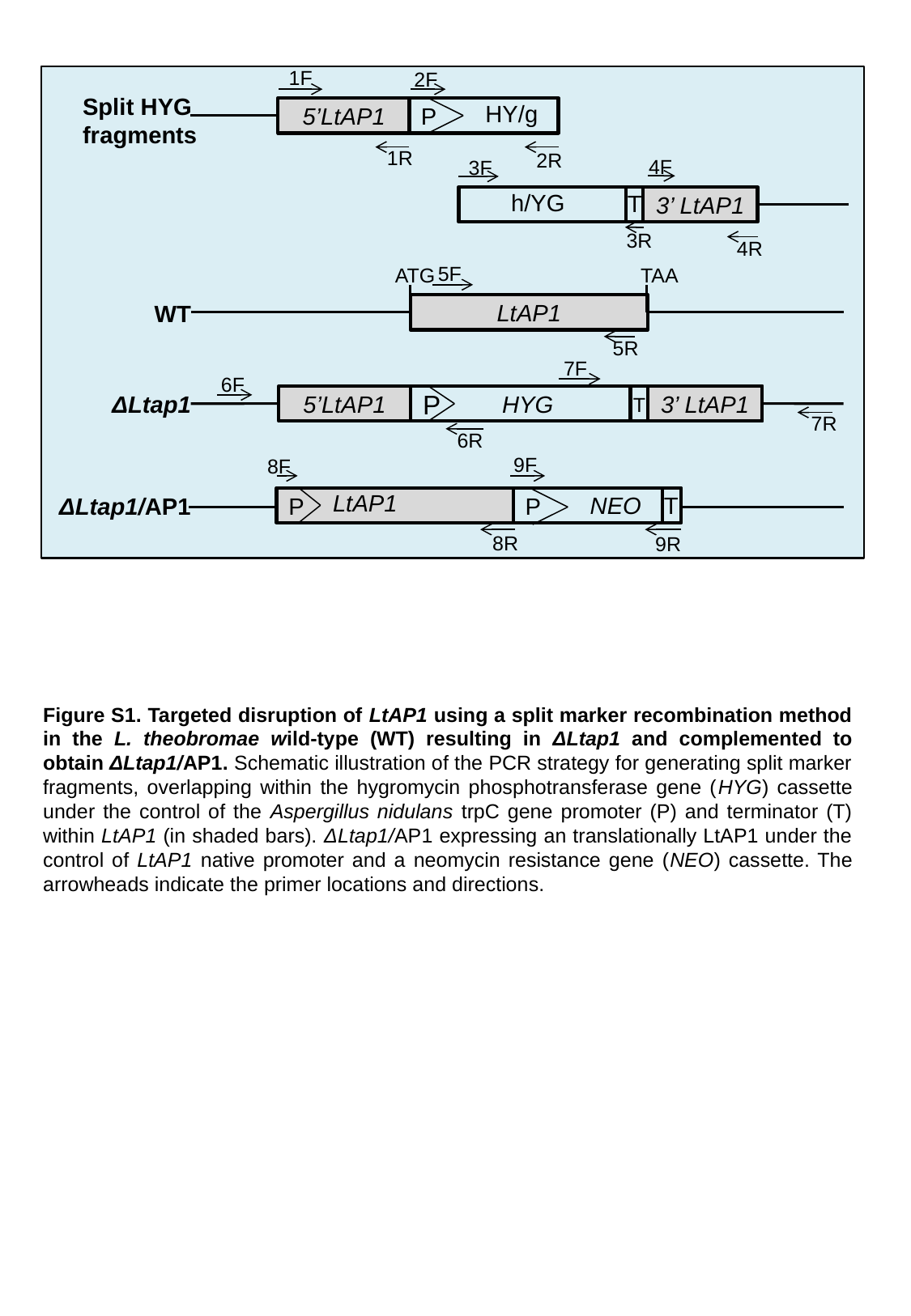

1F
2F
Split HYG
fragments
HY/g
5’LtAP1
P
1R
2R
4F
3F
h/YG
T
3’ LtAP1
3R
4R
5F
ATG
TAA
WT
LtAP1
5R
7F
6F
ΔLtap1
HYG
P
5’LtAP1
3’ LtAP1
T
7R
6R
9F
8F
LtAP1
T
NEO
ΔLtap1/AP1
P
P
8R
9R
Figure S1. Targeted disruption of LtAP1 using a split marker recombination method in the L. theobromae wild-type (WT) resulting in ΔLtap1 and complemented to obtain ΔLtap1/AP1. Schematic illustration of the PCR strategy for generating split marker fragments, overlapping within the hygromycin phosphotransferase gene (HYG) cassette under the control of the Aspergillus nidulans trpC gene promoter (P) and terminator (T) within LtAP1 (in shaded bars). ΔLtap1/AP1 expressing an translationally LtAP1 under the control of LtAP1 native promoter and a neomycin resistance gene (NEO) cassette. The arrowheads indicate the primer locations and directions.

## Slide 2
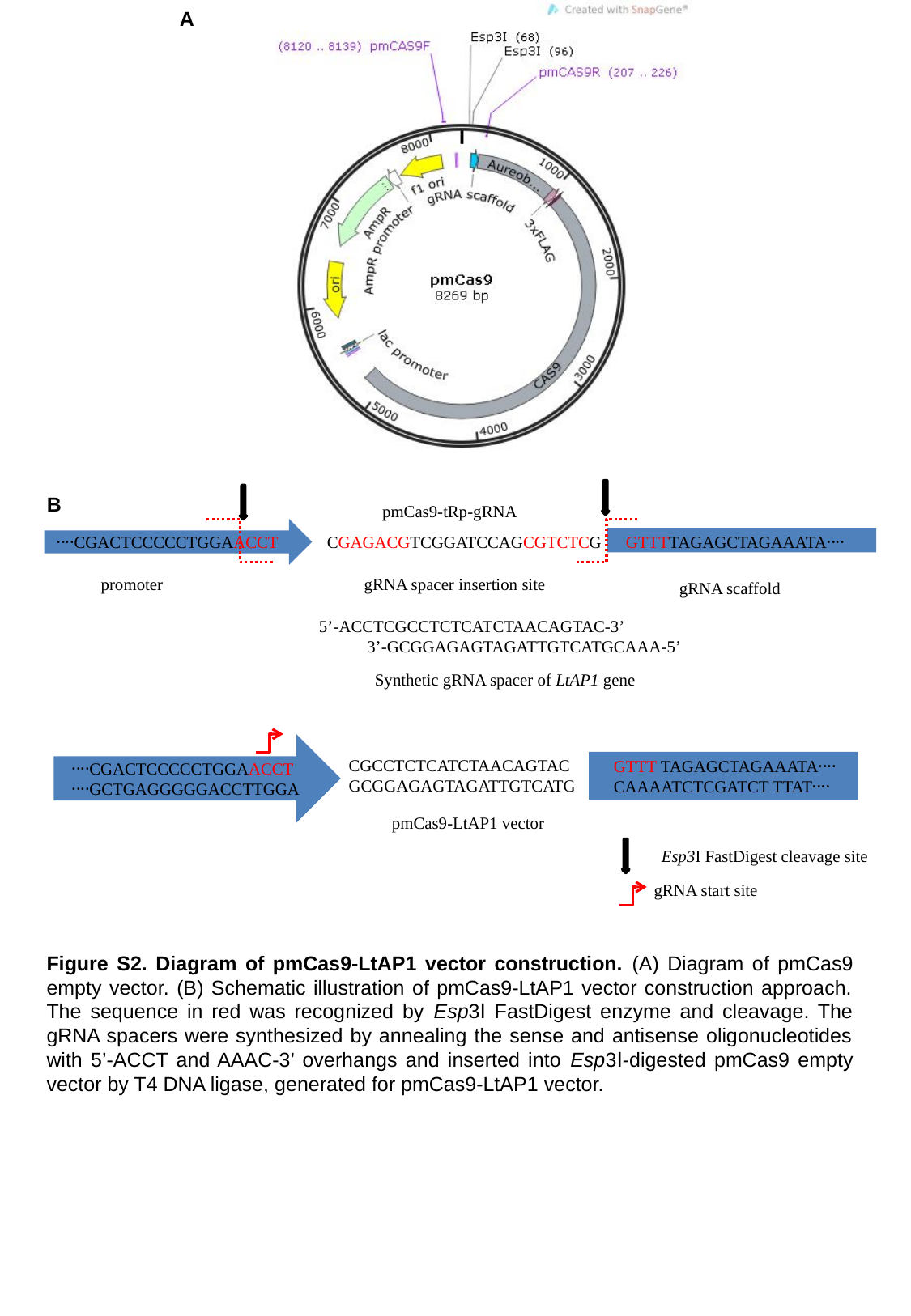

A
B
pmCas9-tRp-gRNA
GTTTTAGAGCTAGAAATA‥‥
‥‥CGACTCCCCCTGGAACCT
CGAGACGTCGGATCCAGCGTCTCG
promoter
gRNA spacer insertion site
gRNA scaffold
5’-ACCTCGCCTCTCATCTAACAGTAC-3’
3’-GCGGAGAGTAGATTGTCATGCAAA-5’
Synthetic gRNA spacer of LtAP1 gene
CGCCTCTCATCTAACAGTAC
GCGGAGAGTAGATTGTCATG
GTTT TAGAGCTAGAAATA‥‥
CAAAATCTCGATCT TTAT‥‥
‥‥CGACTCCCCCTGGAACCT
‥‥GCTGAGGGGGACCTTGGA
pmCas9-LtAP1 vector
Esp3I FastDigest cleavage site
gRNA start site
Figure S2. Diagram of pmCas9-LtAP1 vector construction. (A) Diagram of pmCas9 empty vector. (B) Schematic illustration of pmCas9-LtAP1 vector construction approach. The sequence in red was recognized by Esp3Ⅰ FastDigest enzyme and cleavage. The gRNA spacers were synthesized by annealing the sense and antisense oligonucleotides with 5’-ACCT and AAAC-3’ overhangs and inserted into Esp3Ⅰ-digested pmCas9 empty vector by T4 DNA ligase, generated for pmCas9-LtAP1 vector.

## Slide 3
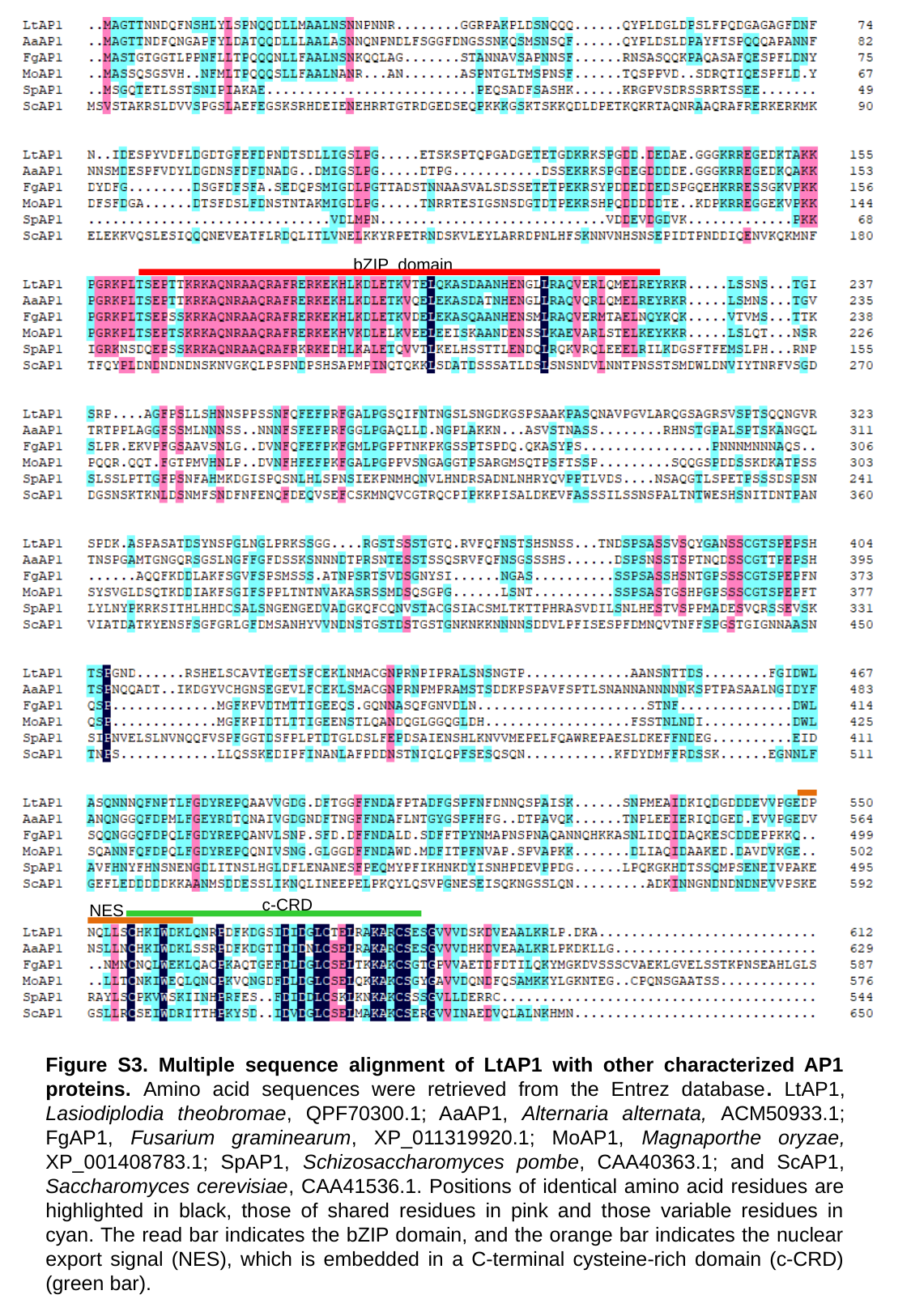

bZIP domain
c-CRD
NES
Figure S3. Multiple sequence alignment of LtAP1 with other characterized AP1 proteins. Amino acid sequences were retrieved from the Entrez database. LtAP1, Lasiodiplodia theobromae, QPF70300.1; AaAP1, Alternaria alternata, ACM50933.1; FgAP1, Fusarium graminearum, XP_011319920.1; MoAP1, Magnaporthe oryzae, XP_001408783.1; SpAP1, Schizosaccharomyces pombe, CAA40363.1; and ScAP1, Saccharomyces cerevisiae, CAA41536.1. Positions of identical amino acid residues are highlighted in black, those of shared residues in pink and those variable residues in cyan. The read bar indicates the bZIP domain, and the orange bar indicates the nuclear export signal (NES), which is embedded in a C-terminal cysteine-rich domain (c-CRD) (green bar).

## Slide 4
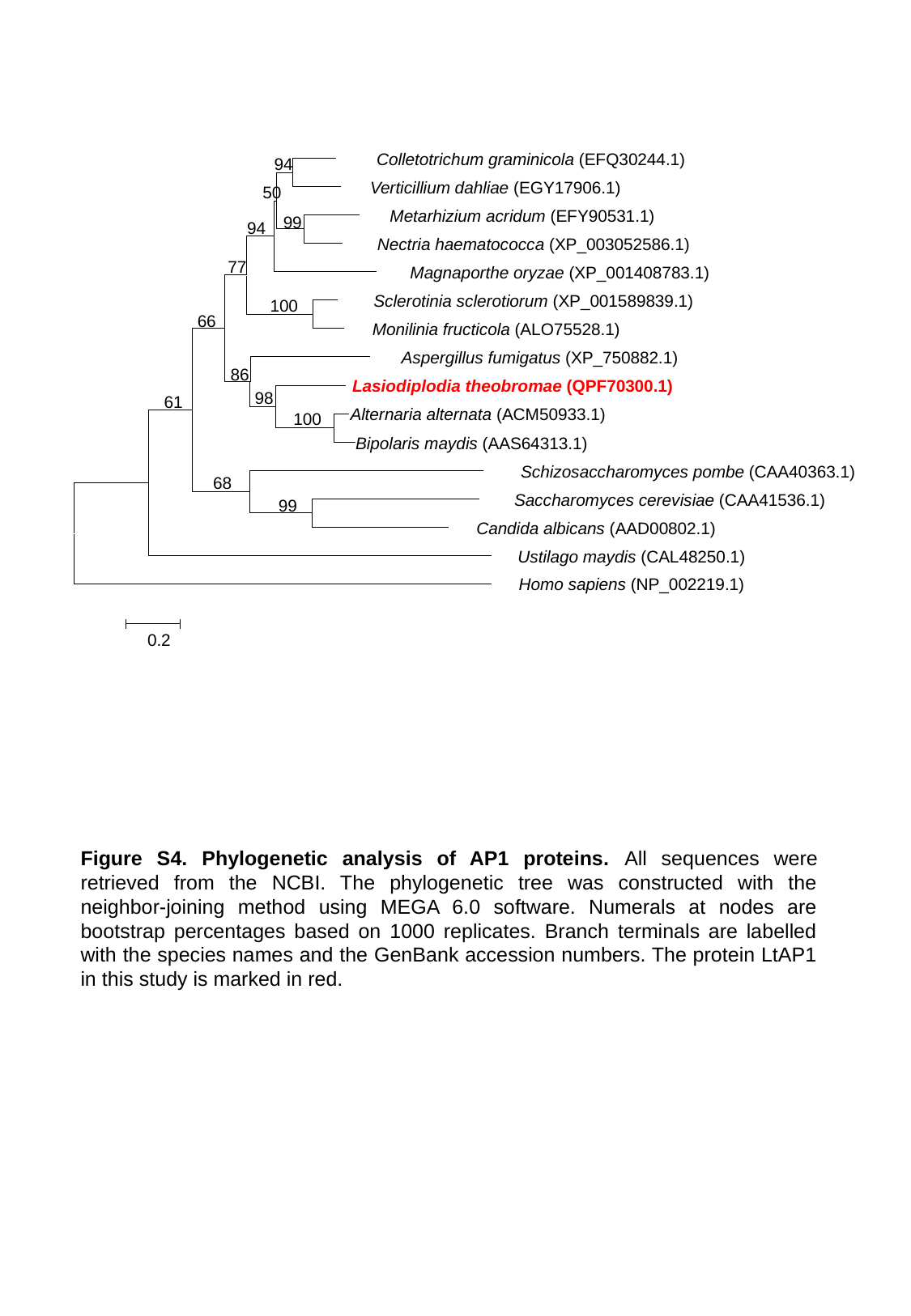

Colletotrichum graminicola (EFQ30244.1)
94
Verticillium dahliae (EGY17906.1)
50
Metarhizium acridum (EFY90531.1)
99
94
Nectria haematococca (XP_003052586.1)
77
Magnaporthe oryzae (XP_001408783.1)
Sclerotinia sclerotiorum (XP_001589839.1)
100
66
Monilinia fructicola (ALO75528.1)
Aspergillus fumigatus (XP_750882.1)
86
 Lasiodiplodia theobromae (QPF70300.1)
98
61
Alternaria alternata (ACM50933.1)
100
Bipolaris maydis (AAS64313.1)
Schizosaccharomyces pombe (CAA40363.1)
68
Saccharomyces cerevisiae (CAA41536.1)
99
Candida albicans (AAD00802.1)
Ustilago maydis (CAL48250.1)
Homo sapiens (NP_002219.1)
0.2
Figure S4. Phylogenetic analysis of AP1 proteins. All sequences were retrieved from the NCBI. The phylogenetic tree was constructed with the neighbor-joining method using MEGA 6.0 software. Numerals at nodes are bootstrap percentages based on 1000 replicates. Branch terminals are labelled with the species names and the GenBank accession numbers. The protein LtAP1 in this study is marked in red.

## Slide 5
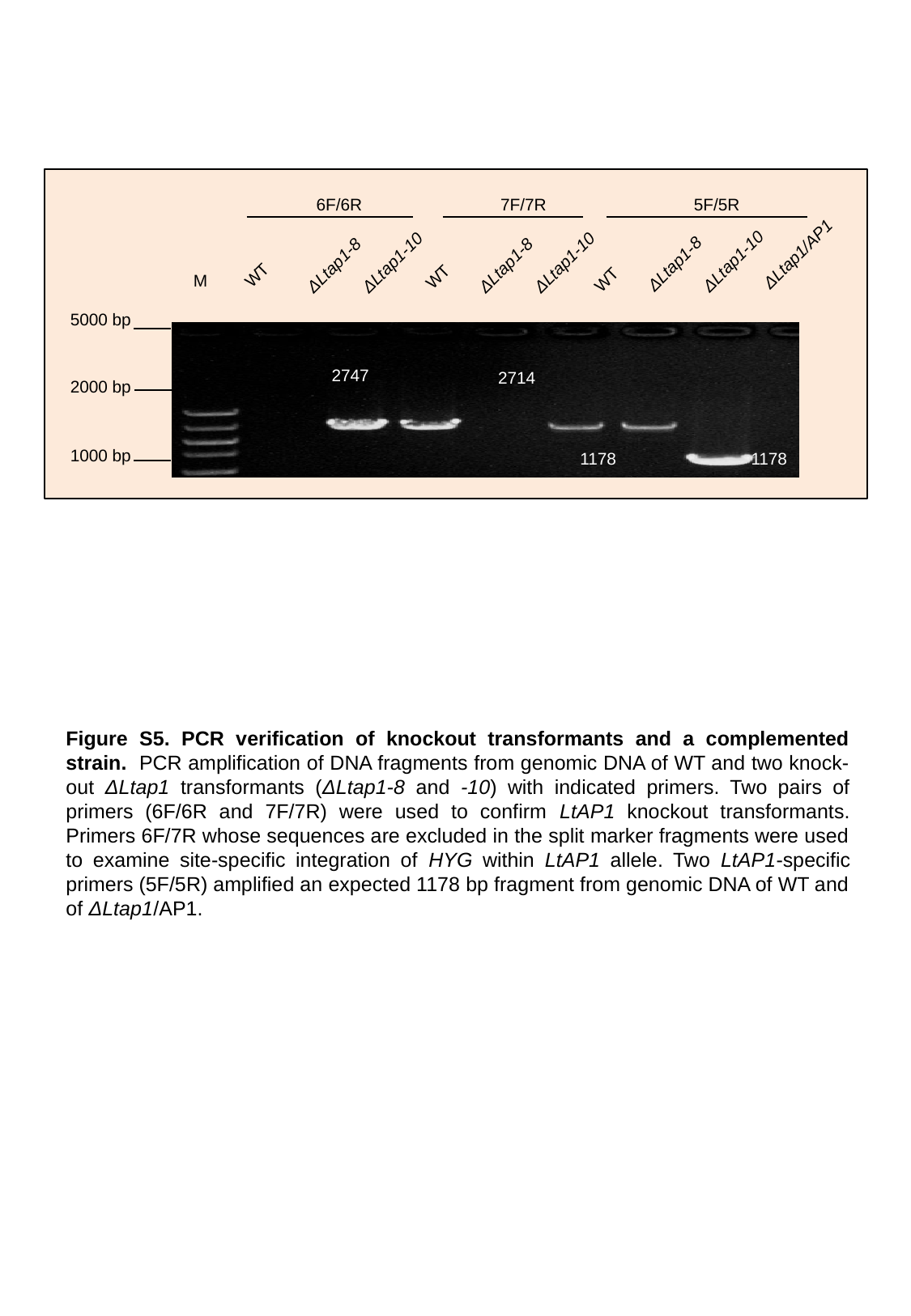

6F/6R
7F/7R
5F/5R
ΔLtap1/AP1
ΔLtap1-10
ΔLtap1-10
ΔLtap1-10
ΔLtap1-8
ΔLtap1-8
ΔLtap1-8
WT
WT
WT
M
5000 bp
2747
2714
2000 bp
1000 bp
1178
1178
Figure S5. PCR verification of knockout transformants and a complemented strain. PCR amplification of DNA fragments from genomic DNA of WT and two knock-out ΔLtap1 transformants (ΔLtap1-8 and -10) with indicated primers. Two pairs of primers (6F/6R and 7F/7R) were used to confirm LtAP1 knockout transformants. Primers 6F/7R whose sequences are excluded in the split marker fragments were used to examine site-specific integration of HYG within LtAP1 allele. Two LtAP1-specific primers (5F/5R) amplified an expected 1178 bp fragment from genomic DNA of WT and of ΔLtap1/AP1.
